# Time-Resolved PIV Measurements and Turbulence Characteristics of Flow Inside an Open-Cell Metal Foam

**DOI:** 10.3390/ma14133566

**Published:** 2021-06-25

**Authors:** Youngwoo Kim, Chanhee Moon, Omid Nematollahi, Hyun Dong Kim, Kyung Chun Kim

**Affiliations:** 1 School of Mechanical Engineering, Pusan National University, Busan 46241, Korea; ywkim@pusan.ac.kr (Y.K.); chmoon@pusan.ac.kr (C.M.); omid@pusan.ac.kr (O.N.); 2 Rolls-Royce and Pusan National University Technology Centre, Pusan National University, Busan 46241, Korea

**Keywords:** open-cell metal foam, flow characteristics, time-resolved PIV, 3D printing, refractive index matching

## Abstract

Open-cell metal foams are porous medium for thermo-fluidic systems. However, their complex geometry makes it difficult to perform time-resolved (TR) measurements inside them. In this study, a TR particle image velocimetry (PIV) method is introduced for use inside open-cell metal foam structures. Stereolithography 3D printing methods and conventional post-processing methods cannot be applied to metal foam structures; therefore, PolyJet 3D printing and post-processing methods were employed to fabricate a transparent metal foam replica. The key to obtaining acceptable transparency in this method is the complete removal of the support material from the printing surfaces. The flow characteristics inside a 10-pore-per-inch (PPI) metal foam were analyzed in which porosity is 0.92 while laminar flow condition is applied to inlet. The flow inside the foam replica is randomly divided and combined by the interconnected pore network. Robust crosswise motion occurs inside foam with approximately 23% bulk speed. Strong influence on transverse motion by metal foam is evident. In addition, span-wise vorticity evolution is similar to the integral time length scale of the stream-wise center plane. The span-wise vorticity fluctuation through the foam arrangement is presented. It is believed that this turbulent characteristic is caused by the interaction of jets that have different flow directions inside the metal foam structure. The finite-time Lyapunov exponent method is employed to visualize the vortex ridges. Fluctuating attracting and repelling material lines are expected to enhance the heat and mass transfer. The results presented in this study could be useful for understanding the flow characteristics inside metal foams.

## 1. Introduction

Flow through porous media is a topic of interest in many disciplines of science and engineering, such as petroleum engineering [1], groundwater hydrology [2], heat exchangers [3], catalyst reactors [4], mixers [5], and filters [6]. Fluid flow through porous media should be studied carefully because the local flow characteristics can affect the global characteristics of the fluid systems [7,8]. Open-cell metal foams are an irregular metallic porous medium [9]. The skeletal part of the metal foam is a Plateau’s border network and involves nodes and struts [10]. The skeletal part forms trabecular-like bone cells [11], which deliver desired geometrical features for thermo-fluidic applications, including high-porosity [12], big specific surface zone [13], twisting flow paths [14], and good strength [15].

Metal foams are fascinating because of their applicability in various fields. Many experimental/ numerical methods have been employed to study flows in metal foam. Flow visualization methods are favorable approaches to understanding the fluid flow physics of metal foams. Studies including lump parameters inherently neglect the localized flow features. Additionally, numerical studies highly require robust experimental results for validation [16].

To date, several works have been done regarding experimental fluid flow visualization. Hwang et al. [17] qualitatively visualized the flow of inlet and outlet of two different metal foams (porosity: 0.7 and 0.9) using smoke-wire. When the porosity was 0.7, the amount of smoke accumulation upstream was larger and so was the size of eddies downstream from the metal foam. The smoke accumulation and eddies may represent the permeability related to the pressure drop. Eggenschwiler et al. [18] investigated the velocity uniformity of the highly asymmetric flow through a honeycomb monolith and a metal foam. The flow through the metal foam showed much higher velocity uniformity than that through the honeycomb monolith. The homogenization of the velocity profile through the metal foams might have been caused by effects of the structure and the relatively high pressure drop.

Hutter et al. [19] investigated the flow and mass transfer in the flow upstream and downstream of three different metal foams with 20, 30, and 45 pores-per-inch (PPI) using particle image velocimetry (PIV) and laser-induced fluorescence (LIF). The flow behind the 20-PPI metal foam had higher turbulent kinetic energy and mixing performance than that behind the 30 and 45-PPI metal foams. This result indicates that the flow behavior in metal foams should be carefully characterized prior to use.

Butscher et al. [20] investigated the flow through a foam-like porous structure (porosity: 0.78) with periodic and uniform cell topology using two-frame PIV and refractive index matching techniques. In their experiment, the jet flowing into the cells was decelerated by the cell geometry, which enhanced the heat and mass transfer. Their results are helpful for understanding metal foam flow, but the high-porosity metal foam structure cannot be printed by stereolithography 3D printing.

Ensemble-averaged flow fields through 4× scale foam were studied by Onstad et al. [21] employing magnetic resonance velocimetry (MRV). A turbulent flow with cell Reynolds of 840 (bulk Reynolds: 7900) was considered when passing through the foam. A strong transverse flow was evident with 20 to 30% of superficial speed. MRV suffers from achieving TR results when flow field is complex and small, such as in metal foam fluid flow.

Regarding the literature review, to the best of the author’s knowledge, a detailed characteristics study of the fluid flow inside metal foams has not been published. Therefore, the flow structure inside of those materials is unclear. Several parameters, including laminar inlet condition, temporal features, and the fluid flow evolution inside foams, should be further investigated. Thus, this study described a detailed method for TR PIV measurement of a transparent foam. The spatiotemporal features of fluid flow through the metal foam were investigated. The outcome of this study can be used for both theoretical or numerical research. Theoretical flow structures, phenomena, and evolution of flow through the metal foams will be known, which helps further implementation of these structures into the new application. Numerically, the results of this study will be a robust case study for validation of computational fluid dynamics (CFD) simulations.

## 2. Materials and Methods

### 2.1. Fabrication and Refractive Index Matching of Transparent Metal Foam Replica

Aluminum foam with 10 PPI (Duocel^®^ Foam, ERG Aerospace Corp., Oakland, CA, USA.) was prepared as a metal foam replica. X-ray tomography with 743 × 740 × 459 voxels was employed to generate a 3D geometry file with spatial resolution of 0.032 mm. In computer-aided design (CAD) software, replica was cropped to dimensions of 10 × 10 × 25 mm. Due to resolution of the printer, the size of the cropped replica was doubled. Then, the final sizes of the model file were 20 × 20 × 50 mm. The detailed geometry information is provided in Table 1.

**Figure 1:**
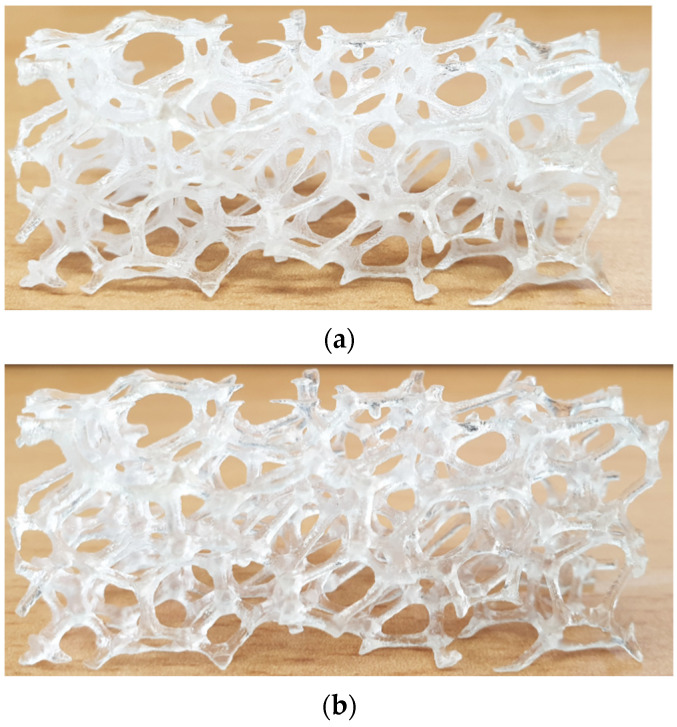
3D printed transparent replica of metal foam (**a**) before and (**b**) after post-processing for refractive index matching.

Two 3D printing techniques that can be used to print transparent porous media are the stereolithography and PolyJet methods. In Stereolithography, the surface of liquid resin is irradiated with a UV laser to cure it. In the PolyJet method, fine resin droplets are jetted onto the model surface and cured by UV lamps. The PolyJet method can print different materials simultaneously. Stereolithography provides better surface roughness of the printed model, but the PolyJet method is suitable for printing metal foam structures because the struts of the metal foam need support material when they are printed. Figure 1a shows the replica printed by PolyJet 3D printer. Printer materials were Vero Clear resin and water soluble support material.

Achieving an optically clear surface is crucial; therefore, before starting the experimental campaign, a series of post-processing actions should be performed. This procedure includes:

1. The support material must be removed completely since it is not transparent. This was done using a water jet after soaking the model for half a day.
2. Wet sanding must be done to obtain the best transparency. The sanding was started from a coarse sand to finer one. All the surfaces were sanded, and each time, the model was inspected under a light source to recognize the sandpaper size change where surface defects were no longer visible after last sanding pass. Each sanding step was done perpendicular to the previous one.
3. The printed model was then placed in a water channel with water circulating at ambient temperature for 48 h.
4. For the final step, polishing was done by a polishing substantial to achieve a glossy high-quality surface.

The printing orientation was also considered as [20,22]. Figure 1b shows the 3D printed transparent foam after the completion of every process. As can be seen after post-processing procedures, a glossy surface is achieved.

**Figure 2:**
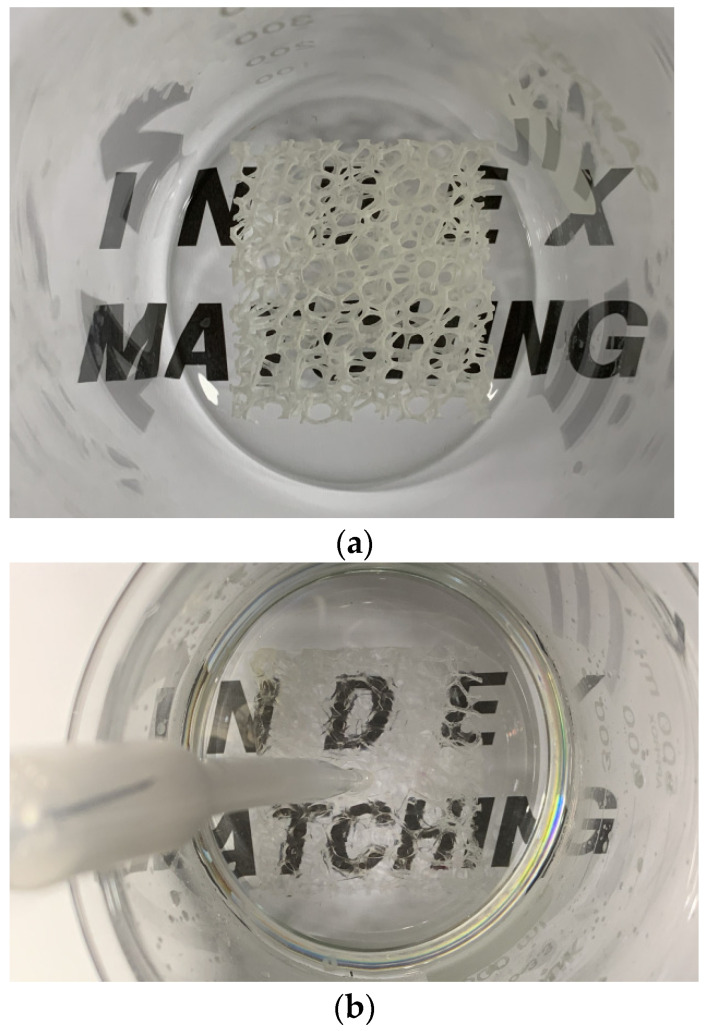
Metal foam replica without (**a**) and with (**b**) index matching.

The main issue regarding the flow-field measurement inside a foam replica is to avoid optical distortion. Therefore, the refractive index should be studied. The refractive index of Vero Clear material is 1.515 for a wavelength of 532 nm. A refractive index matched (RIM) solution based on NaI was provided using the solution formula developed by Gallagher et al. [23] (60.2 wt% NaI, 32.4 wt% water, and 7.36 wt% glycerin). The grades of reagents were higher than extra pure. Water was degassed in a vacuum chamber before making RIM. In addition, the RIM was degassed by increasing the temperature to 40 °C and then cooled down to room temperature. Sodium thiosulfate (0.1 wt%) was added to avoid the discoloration [24] caused by NaI reacting with oxygen to form triiodide ions. In addition, it is recommended to store the solution in a glass container, because sodium thiosulfate reacts with some metals, such as aluminum and stainless steel, to form complexes. To demonstrate the suitability of RIM, Butscher, Hutter, Kuhn, and von Rohr 2012 utilized a sample text below the specimen to present the RIM, while Aycock et al. 2017 employed a sample gride under the replica to show the RIM in a qualitative manner. In the current study, the method of Butscher, Hutter, Kuhn, and von Rohr 2012 was applied to confirm the RIM. Figure 2a,b presents the metal foam replica without and with index matching, respectively. As evidenced, it can be seen that using above procedures led to a suitable-matched refractive index in comparison.

### 2.2. Experiment Test Section

**Figure 3:**
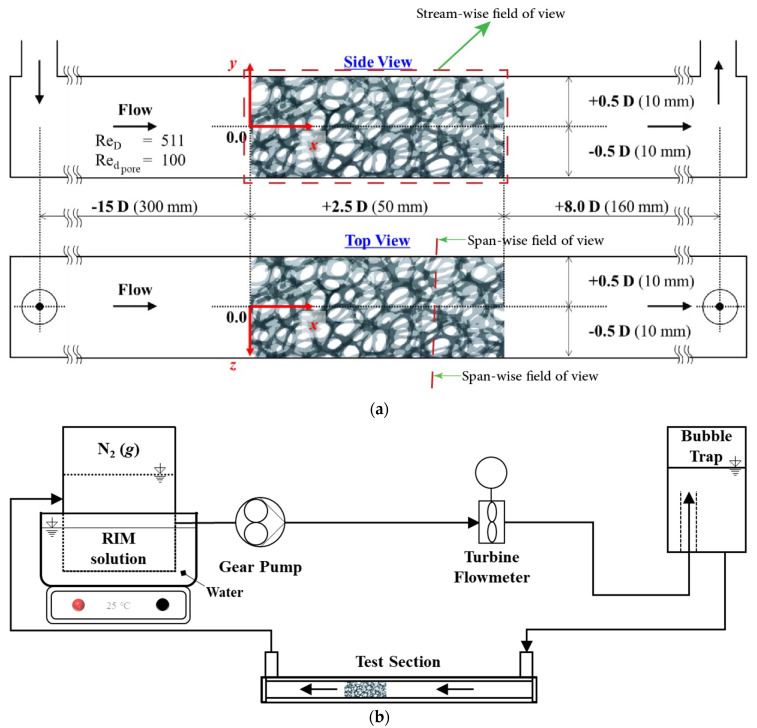
Experimental setup configuration: (**a**) test section details; (**b**) overall setup configuration.

A square acrylic duct was manufactured (20 mm × 20 mm × 500 mm), as shown in Figure 3a. Non-dimensional sizes based on the duct width, which, from now on, will becalled diameter (D) for a better wording to compare with previous works, are indicated. Inlet and outlet of the duct are located on the upper side of the duct to see span-wise fluid flow. The model was placed at a length of 15 D from inlet to remove the entrance length effects.

A loop was arranged to allow the RIM to flow over the test section. The test loop included gear pump, RIM container, bubble trap, flowmeter, and test section. Silicon hose connected the components. To maintain the RIM solution temperature, a constant temperature hot plate of 25 °C was located under the solution container. In addition, the container was partly immersed in a water bath.

**Figure 4:**
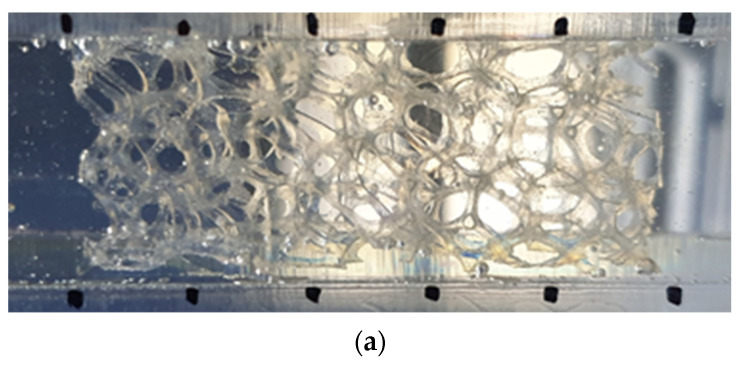

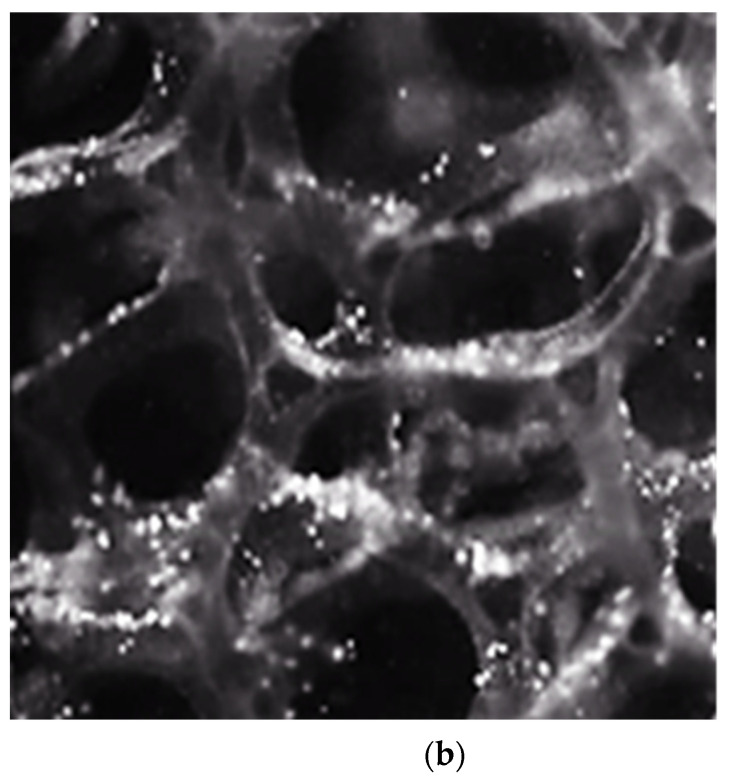
Test section with refractive-index-matched solution and transparency of the 3D printed model: (**a**) without laser illumination; (**b**) with laser illumination.

Figure 4a shows the test section filled with the prepared RIM solution. It is practically impossible to match the refractive index completely, so the many edges of the metal foam replica are still shown. However, it has acceptable transparency, as shown in Figure 4b.

### 2.3. PIV Measurement Setup

**Figure 5:**
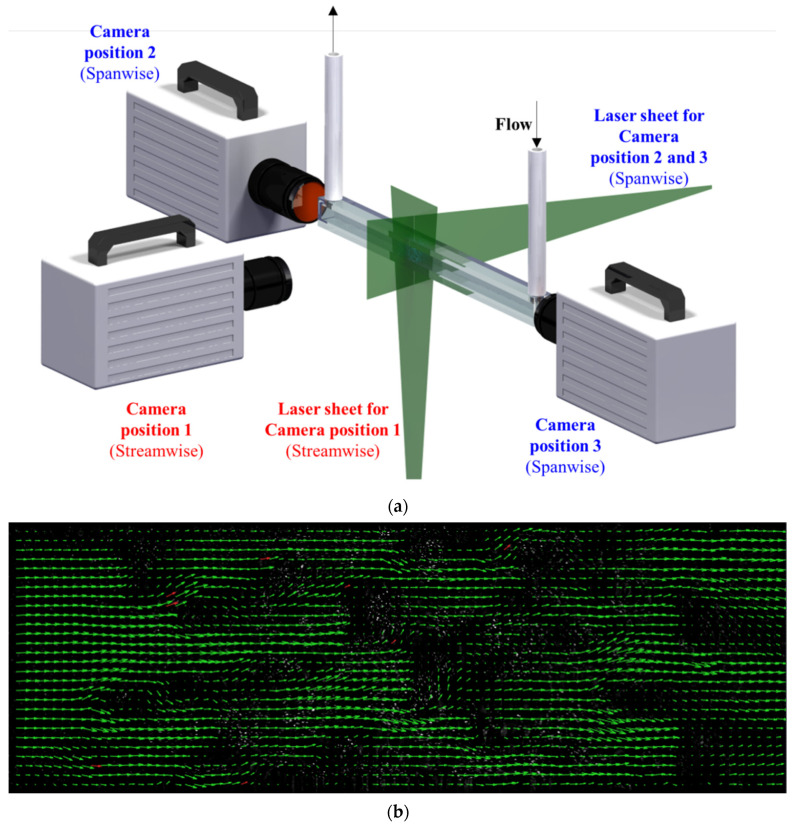
(**a**) PIV measurement setup; (**b**) instantaneous velocity vectors (stream-wise—position 1).

A CMOS high-speed camera (FastCam SA1.1, Photron, 1k × 1k pixels) and a 5 W, 532 nm continuous wave laser were employed to conduct TR 2-D PIV. Figure 5a shows the positions of the camera and laser sheet. Position 1 was used to observe stream-wise planes, while positions 2 and 3 were used to observe span-wise planes. The equivalent diameter of the struts was less than 1 mm, so the laser sheets thickness was fixed to 0.8 mm. Fluorescent polymer particles (1–20 µm) coated with rhodamine B were used as the tracer particles.

Choosing the best time spacing between image pairs (camera framer rate) was dependent to the camera sensor size (pixels), real field of view size (physical sizes), and flow field velocity. Based on these parameters, the frame rate of the camera was set to 750 fps, considering the flow velocity in the pores. Greyscale images with 8-bit depth and 10,000 frames were captured and processed by the TR PIV algorithm. The background was subtracted using the POD filter developed by Mendez et al. [25]. The velocity vector fields were obtained by the fast Fourier transform multi-pass algorithm [26]. The interrogation sizes of the passes were 64 × 64 pixels, 32 × 32 pixels, and 24 × 24 pixels.

The displacement of the particles was about 1/6 of the final size of the interrogation area [27]. Each interrogation area was overlapped by 50%. Based on the displacement information from the overlapped borders and corners of the interrogation area, the interrogation area was deformed by spline interpolation to interpolate the data between the passes. A two-dimensional Gaussian function was used to find the peak intensity of the correlation matrix [28]. A local median filter was used to validate the velocity vectors [29]. A sample of the stream-wise instantaneous velocity vector images is shown in Figure 5b. The velocity vectors that were filtered by the local median filter are indicated in red. The evidence of suitable camera frame rate was that there were no bad vectors or unusual vectors in Figure 5b; therefore, we could be sure that the selected fps was considered enough to capture the flow field characteristics.

### 2.4. Preliminary Experiments

**Figure 6:**
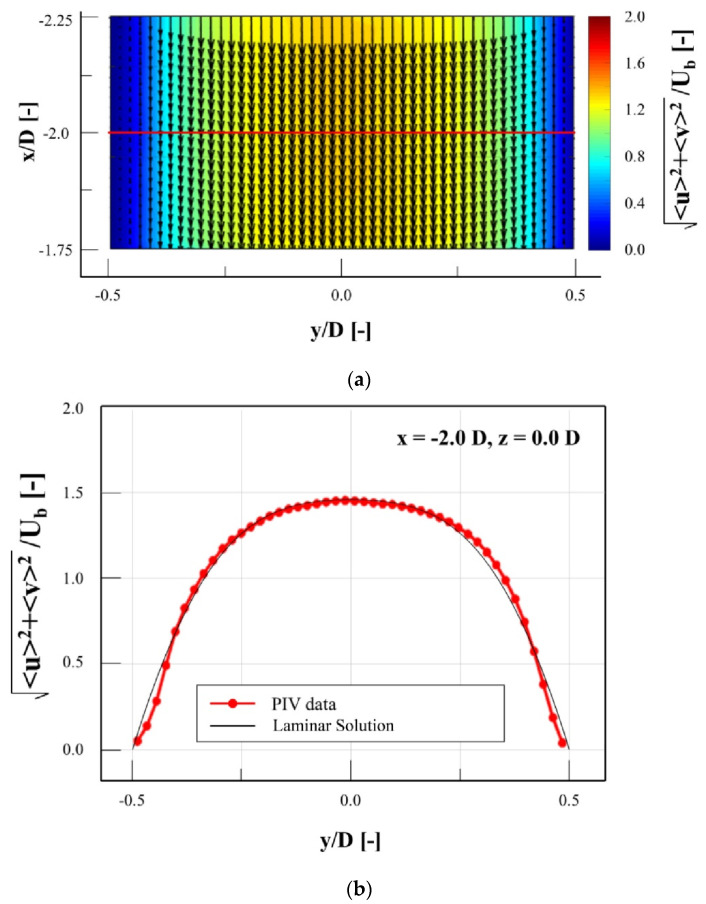
Inlet flow conditions: (**a**) velocity contour superimposed with velocity vectors upstream of the metal foam replica; (**b**) the comparison of velocity profiles from PIV measurements (experimental) and laminar solution.

Before starting the experimental campaign, a semi-validation of setup was done to show how the experimental setup and FPS were suitable to capture the flow structure. In addition, it can be used to demonstrate the development condition of the velocity profile at the inlet of porous media, as in [20]. The bulk velocity ($U_{b}$) was calculated from the volume flow rate as 0.82 ± 0.01 m/s within a 95% confidence level. The Reynolds numbers based on channels hydraulic diameter was 511, while the Reynolds number based on pore diameter was 100. These numbers prove that the flow regime was laminar; however, based on the Reynolds number of the pore diameter following recent publications [30,31,32], the flow regime was categorized as unsteady laminar, where the laminar wake oscillates. In addition, a bubble trap was installed to minimize the inflow of bubbles into the test section. Figure 6a shows the velocity contour superimposed with velocity vectors upstream of the metal foam replica for instantaneous velocity contour (x: −1.75 D~−2.25 D, y: −0.5 D~0.5 D, z: 0 D).

The stream-wise velocity U can be defined as:

(1) $$U=\langle u(x,y,z)\rangle +u^{\prime }\left(x,y,z,t\right)$$

where 〈u(x,y,z)〉 is the mean velocity, and $u^{\prime }\left(x,y,z,t\right)$ is the fluctuation velocity. The other velocity components can also be defined in the same manner. The velocity magnitude diagram at (−2 D, −0.5 D~0.5 D, 0 D) is shown in Figure 6b. This location is shown as a red line in Figure 6a. The velocity profile shows good agreement with the numerical solution for the laminar flow in the same square duct. This result shows that the flow upstream of the metal foam replica is laminar. In addition, it demonstrates that the flow at the inlet of the porous material is fully developed. However, a little deviation is obvious, which can be an evidence of unsteadiness, which was previously discussed as unsteady laminar flow.

## 3. Results and Discussion

### 3.1. Velocity Magnitude

**Figure 7:**
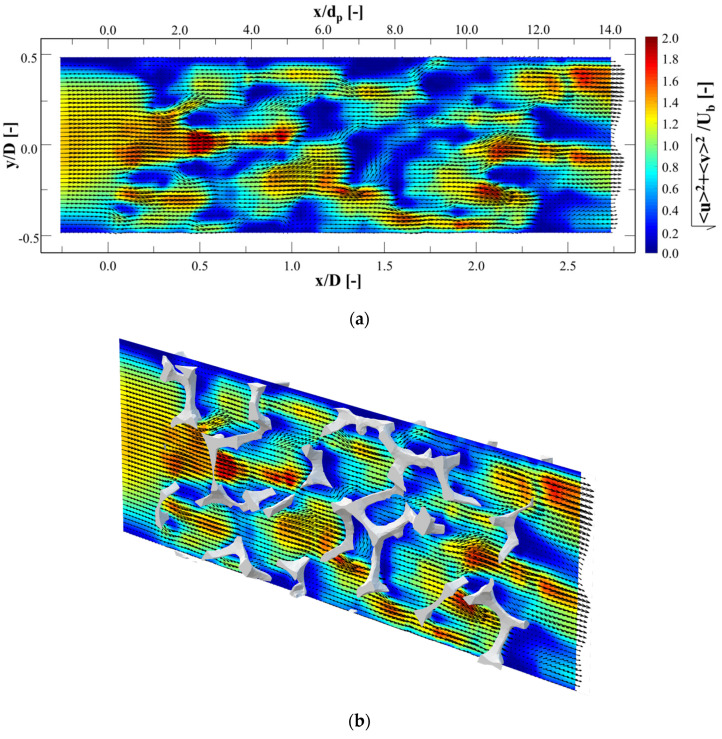
Contours of mean velocity magnitude on the plane at *z* = 0 D with superimposed vector field: (**a**) without and (**b**) with overlay of metal foam structure.

Figure 7a presents the mean velocity contours of *u* and *v* at *z* = 0, which is superimposed with velocity vectors. The direction of flow is from left to right. x-direction distance is non-dimensionalized by both pore and channel diameters in the top and bottom of figure, respectively. The channel diameter is employed to non-dimensionalized *y*-direction distance, and the mean velocity is non-dimensionalized by the bulk velocity. Figure 7b shows the mean velocity contours overlaid with the metal foam structure. The solid model was cropped by *z* = ±0.1 D.

The main flow upstream of the foam was divided to two or three streams by the foam pores when the main flow met the foam structure for first time. Several jets were made at *x* = 0 to 0.25 D. After passing through the cells, the jets slowed; however, new jets were simultaneously shaped by other pores. This conversion from earlier jets to new ones continued steadily. This separation and combination through the foam led to well-mixing of fluid. Considering that the current study performed a 2D measurement, the number of these separations and combinations will be greater.

The separation regions behind the struts should be considered since they affect pressure drop. However, Figure 7 does not present out-of-plane velocity, but separation performance is in agreement with Onstad et al. [21]. The size of the separation area is supposed to depend on the metal foam strut shape. The strut’s shape is circular or triangular depending on porosity, according to Plateau’s law, which describes higher porosity in a more triangular strut shape. The current study was performed on a triangular strut shape. Moon et al. [33] studied the effect of shape of strut in Kelvin cells on heat transfer and pressure drop.

### 3.2. Mean Velocity Profile

**Figure 8:**
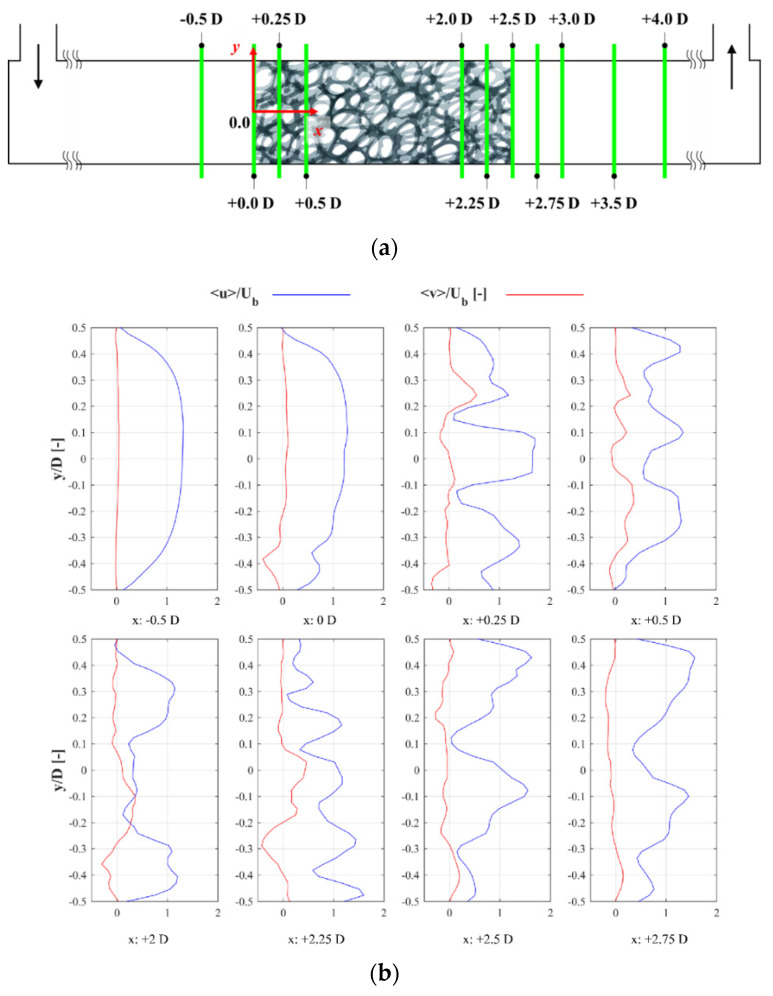
(**a**) Mean velocity plot location in the test section; (**b**) mean velocity profiles.

To extract the velocity profiles on the span-wise flow, the sheet laser was changed according to Figure 8a. Figure 8b shows the velocity profiles on the plane at *z* = 0 D. The blue line and red line show the normalized stream-wise velocity component <u>/$U_{b}$ and normalized transverse velocity component <v>/$U_{b}$, respectively. The peaks of <u>/$U_{b}$ in each part of Figure 8 represent the jet formed by a pore. When main stream met the foam structure at Figure 8b, the <u>/$U_{b}$ profile began to be influenced by the flow resistance generated by the metal foam structure, while at *x* = 0.25 D, multiple jet-like profiles occurred inside the structure. The number of jets on the plane at *z* = 0 D varied from two to five in different sections due to foam pore numbers.

It is evident that the main change occurred in <v>/$U_{b}$ in comparison with <u>/$U_{b}$, which was representative of the jet direction since <u>/$U_{b}$ magnitudes are not significant. However, both of them should be considered to determine jet direction. The positive peak indicated the + *y*-direction, and the negative peak indicated the ™y-direction. Valleys indicated that the flow was parallel to the *x*-axis or low-velocity region. When <v>/$U_{b}$ was averaged through the foam replica, <v>/$U_{b}$ was around 23%, which is quite similar to the 20–30% results of Onstad et al. [21]. This similarity should be considered precisely since Reynolds number in this work was considerably lower than Onstad et al. [21]. This demonstrates that the advection through the foam model is predominantly affected by replica configuration, which is in agreement with Onstad et al. [21], who state that significant transverse movement may happen during laminar conditions.

### 3.3. Mean Vorticity

**Figure 9:**
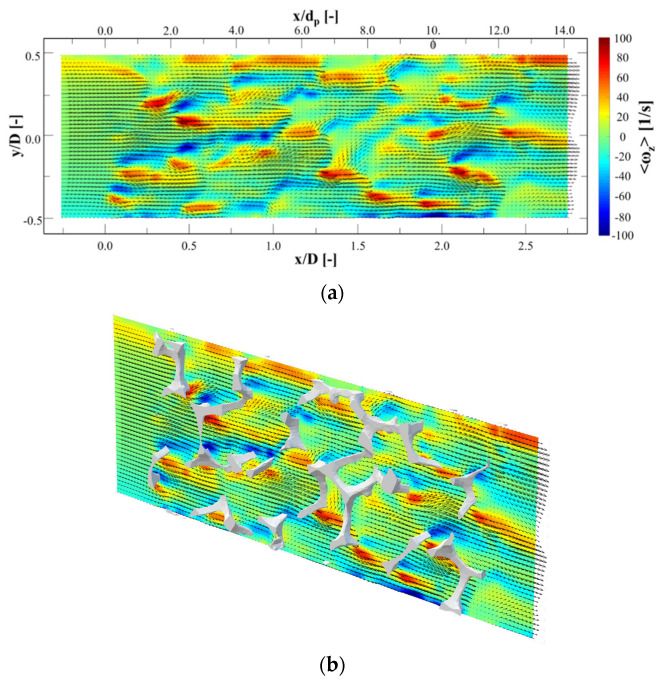
Contours of vorticity at *z* = 0 D with superimposed vector fields: (**a**) without and (**b**) with overlay of metal foam structure.

Figure 9a,b show the contours of the mean vorticity $\langle \omega_{z}\rangle$ on the plane at *z* = 0 D. The out-of-plane vorticity $\langle \omega_{z}\rangle$ in this plane can be defined as:

(2) $$\omega_{z}=\left(\frac{dv}{dx}-\frac{du}{dy}\right)$$

**Figure 10:**
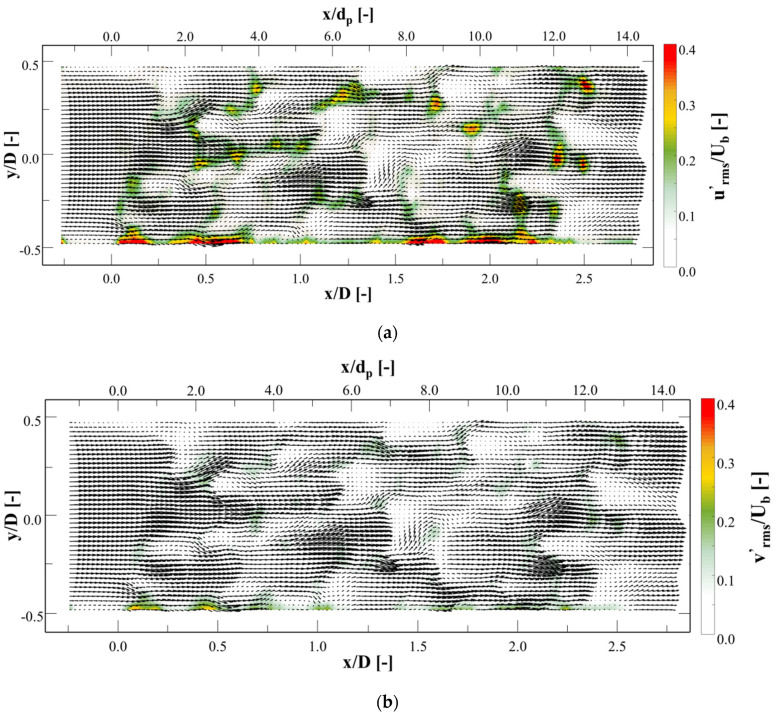
Contours of RMS fluctuation velocities at *z* = 0 D with superimposed velocity vectors: (**a**) stream-wise (*x*-direction) component and (**b**) transverse (*y*-direction) component.

The mean velocity vector field is superimposed on the vorticity contours. It can clearly be seen that many shear layers are formed by the metal foam structures, and these layers are disturbed by the structures and other jets. This increases the flow instability and causes velocity fluctuation. Figure 10a,b show the root-mean-squared (RMS) fluctuation velocity $u_{rms}^{\prime }$ created through the foam, which can be defined as:

(3) $$u_{rms}^{\prime }=\sqrt{\frac{{u_{1}^{\prime }}^{2}+{u_{2}^{\prime }}^{2}+{u_{l}^{\prime }}^{2}}{l}}$$

where ${u_{l}^{\prime }}^{2}$ refers to the fluctuating part of velocity, when instantaneous velocity vector u is decomposed into a mean velocity $\bar{u}$ and a fluctuating part $\overset{´}{u}$ ($u=\bar{u}+\overset{´}{u})$, while l refers to 1 of 10,000 captured frames. The RMS fluctuation of the transverse velocity ($v_{rms}^{\prime }$) can be defined in the same manner.

At the inlet of the foam model, $u_{rms}^{\prime }/U_{b}$ and $v_{rms}^{\prime }/U_{b}$ were near zero, but increased to 0.1–0.4 through the foam model. After leaving the foam model, they approached zero once more. The velocity fluctuated in several areas, including the pore outlet, jet junction, and behind the struts. This demonstrates that the oscillation might be produced by transverse movements created by the foam configuration or unstable shear layers through the interaction of jets. However, further investigation of the origin of these phenomena is crucial.

### 3.4. Integral Time Scale and Length Scale

The integral time and length scales can be calculated for better understanding. The temporal auto-correlation function ($\rho_{u^{\prime }}$) for the stream-wise velocity component $u^{\prime }$ is defined as [34]:

(4) $$\rho_{u^{\prime }}\left(x,y,z,\tau \right)=\frac{u^{\prime }\left(x,y,z,t\right)u^{\prime }\left(x,y,z,t+\tau \right)}{\langle u^{\prime }\left(x,y,z,t\right)u^{\prime }\left(x,y,z,t\right)\rangle }$$

where *τ* is the time lag. The integral time scale ($T_{u^{\prime }}$) of the stream-wise velocity component $u^{\prime }$ is defined as:

(5) $$T_{u^{\prime }}\left(x,y,z\right)=\int_{0}^{n}\rho_{u^{\prime }}\left(\tau \right)dt$$

where *n* is the time when $\rho_{u^{\prime }}$ reaches the first zero. In this study, *n* is set to the point where $\rho_{u^{\prime }}$ reaches a sufficiently low value (0.05) for practical purposes [35]. Considering the Taylor hypothesis, the integral length scale ($L_{u^{\prime }}$) can be defined as [34]:

(6) $$L_{u^{\prime }}\left(x,y,z\right)=T_{u^{\prime }}\left(x,y,z\right)\langle u\left(x,y,z\right)\rangle$$

**Figure 11:**
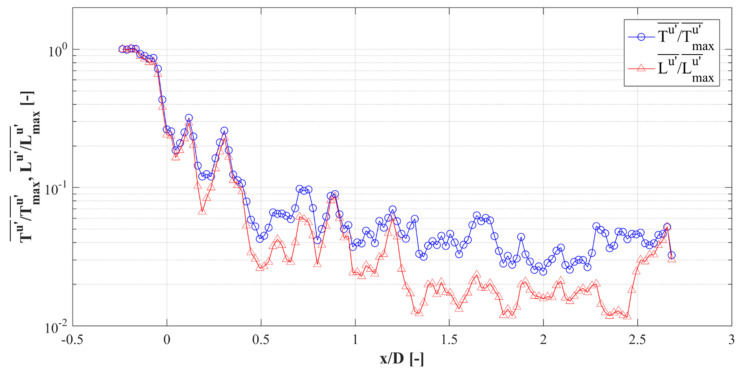
Integral time scale and length scale.

Figure 11 shows the calculated integral time and length scales of $u^{\prime }\left(t\right)$ on the plane at *z* = 0 D. The scales were normalized by their maximum values. The bar indicates the line average for the *y*-axis. The integral time scale rapidly decreases logarithmically at an early stage from 0.5 D to 2.25 D. The integral time scale increases slightly from the outlet region of the metal foam at *x* = 2.0 D–2.25 D. The integral length scale shows a similar tendency to the integral time scale. These results show that the flow gradually becomes complex as it passes through the metal foam structure, which may be an effect of the evolving transverse motion in the structure.

### 3.5. Span-Wise Vorticity

**Figure 12:**
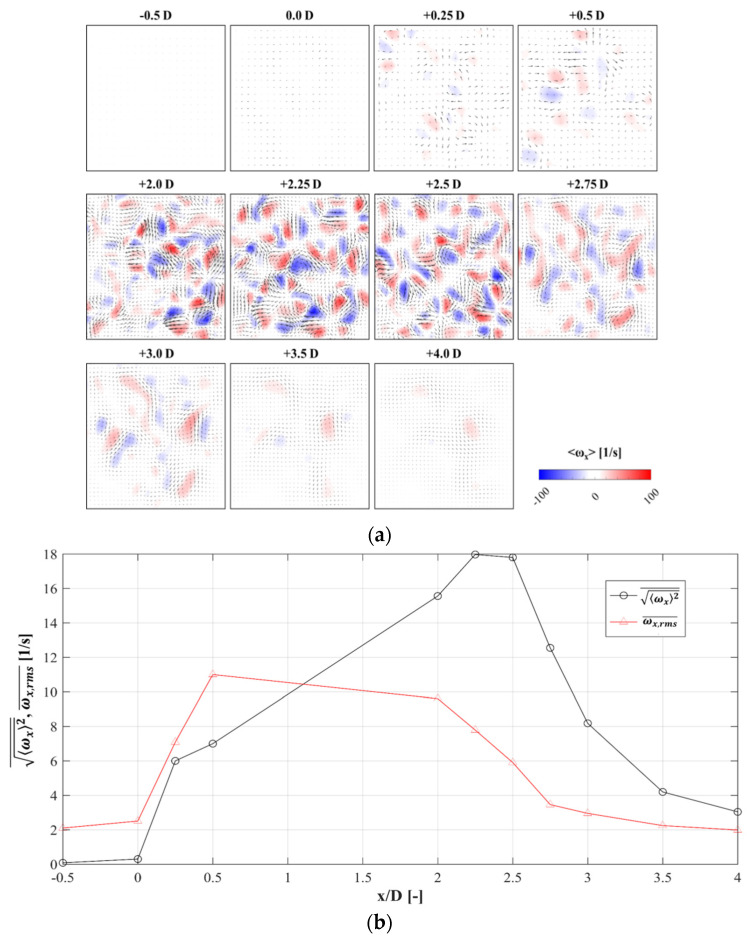
Evolution of span-wise vorticity: (**a**) span-wise vorticity at the positions, and (**b**) spatially-averaged vorticity magnitude—RMS fluctuation values.

**Figure 13:**
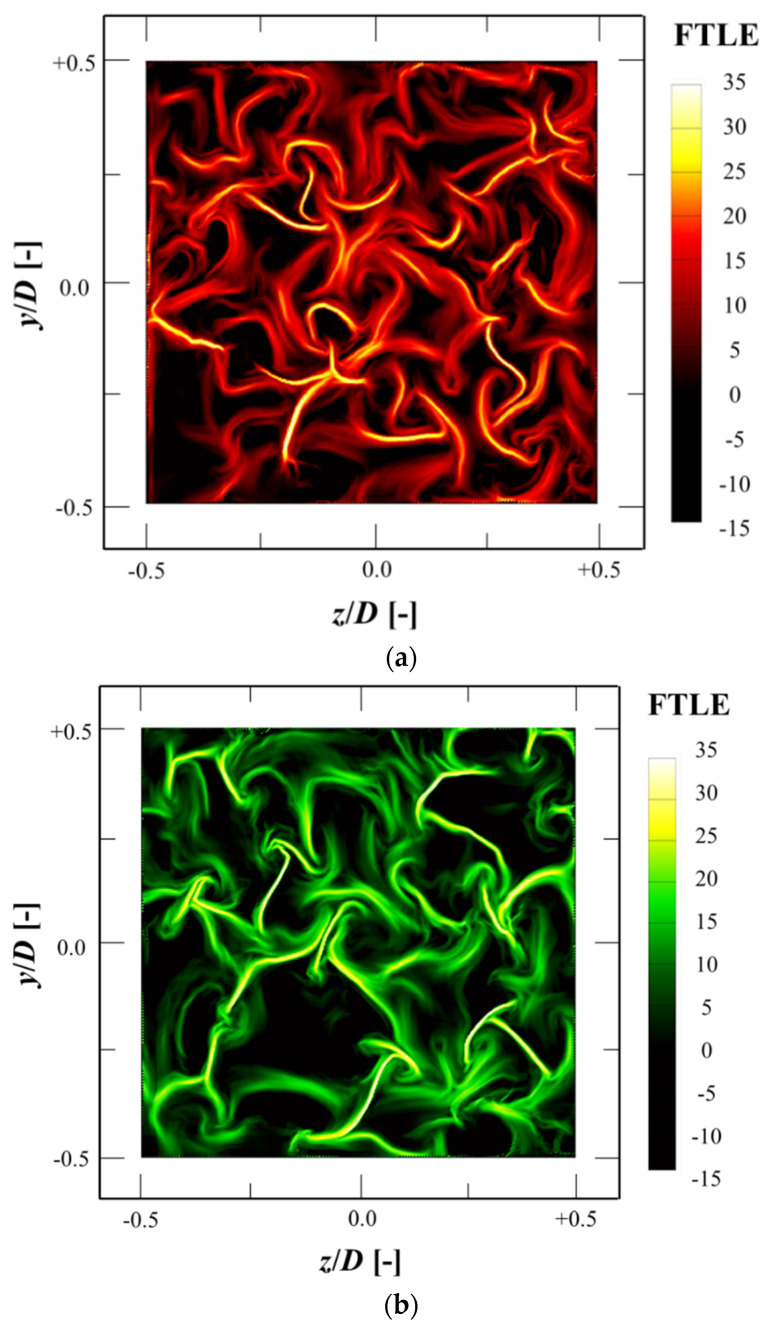
Contours of (**a**) forward and (**b**) backward finite-time Lyapunov exponent (FTLE) at *x* = 2.25 D.

Figure 12 shows the mean vorticity field to illustrate the evolution of the stream-wise vortices through the metal foam structures. The positions of the laser sheet are the same as in Figure 8a. To capture the images, both positions of 2 and 3 were used to capture sharper images. However, it was possible to take images from one side. Sections from −0.5 D to +0.5 D were captured from position 3, while the others were taken from position 2. In Figure 13a, there was no stream-wise vortex at *x* = −0.5 D and 0 D. At *x* = 0.25 D, several counter-rotating stream-wise vortices were generated. At *x* = 2.0 D, the number of vortices was highly increased. At *x* = 2.25 D and 2.5 D, the number and vorticity of vortices appeared similar to each other. At *x* = 2.75 D, these vortices began to dissipate and lose momentum. At *x* = 4.0 D, the vortices had almost dissipated completely. The tendency of the vortex evolution was similar to that of the integral time scale in Figure 11.

The spatially-averaged vorticity magnitude ($\bar{\sqrt{\omega_{x}{}^{2}}}$) and span-wise planes RMS vorticity fluctuation ($\bar{\omega_{x,rms}^{\prime }}$) are revealed in Figure 12b. Vorticity fluctuation is one of the important characteristics of turbulence. The value of $\bar{\sqrt{\omega_{x}{}^{2}}}$ increased while passing through the foam model and then dissipated to the first state once downstream of the foam. $\bar{\omega_{x,rms}^{\prime }}$ increased suddenly in the entrance of the foam model then maintained constant inside the metal foam structure. It is believed that this turbulent characteristic wass caused by the interaction of jets, which have different flow directions inside the metal foam configuration. Interestingly, at *x* = 2.0 D, $\bar{\omega_{x,rms}^{\prime }}$ quickly decreased, and then it slowly decreased through *x* = 2.75–4 D, downstream of the foam replica. $\bar{\omega_{x,rms}^{\prime }}$ quickly decreased during *x* = 2.0–2.5. Downstream of the model, a flow separation zone was formed, which decreased the static pressure.

### 3.6. Finite-Time Lyapunov Exponent (FTLE)

The finite-time Lyapunov exponent (FTLE) method was used to identify the vortical structures of smooth time-dependent velocity fields. FTLE obtains Lagrangian coherent structures (LCSs) by measuring the expansion rate of two neighboring particles during a finite time. FTLE integration during $T_{F}$ is defined as [36]:

(7) $${\mathrm{FTLE}}_{T_{F}}\left(X_{0},\;t_{0}\right)=\frac{1}{\left|T_{F}\right|}\ln\sqrt{\lambda_{max}\left(C_{t_{0}}^{t_{0}+T_{F}}\left(X_{0}\right)\right)}$$

where $T_{F}$ is the integration time, and $\left(X_{0},\;t_{0}\right)$ is the initial position of the tracking fluid particle. $\lambda_{max}\left(C_{t_{0}}^{t_{0}+T_{F}}\left(X_{0}\right)\right)$ is the largest singular value of the Cauchy–Green deformation tensor. The Cauchy–Green deformation tensor ($C_{t_{0}}^{t_{0}+T_{F}}\left(X_{0}\right)$) is expressed as [36]:

(8) $$C_{t_{0}}^{t_{0}+T_{F}}\left(X_{0}\right)={\left[\nabla F_{t_{0}}^{t_{0}+T_{F}}\left(X_{0}\right)\right]}^{T}\cdot \nabla F_{t_{0}}^{t_{0}+T_{F}}\left(X_{0}\right)$$

where $\nabla F_{t_{0}}^{t_{0}+T_{F}}\left(X_{0}\right):X\left(t_{0}\right)\to X\left(t_{0}+T_{F}\right)$ is the flow map, and the superscript *T* is the transpose operator.

$T_{F}$ was set to +1 s and −1 s for the forward and backward FTLE, respectively. The forward FTLE indicates the repelling material line for $T_{F}$ < 0, and the backward FTLE indicates the attracting material line for $T_{F}$ > 0. The grid resolution is 0.1 mm × 0.1 mm.

Figure 13a,b show the contours of the forward and backward FTLE fields at *x* = 2.25 D. Surprisingly, many ridges of the vortical structures were identified. Both the attracting and the repelling material lines were considered to contribute to the heat and mass transfer inside the metal foam structure. The FTLE ridges that had lower integration values could be regarded as fluctuating regions. These regions were expected to contribute greatly to the heat and mass transfer by helping the mechanical mixing by the metal foam structure. This result is consistent with the vorticity fluctuation shown in Figure 13. This also provides evidence of the metal foam causing considerable flow disturbances, even at a relatively low Reynolds number.

## 4. Conclusions

A methodology has been presented for TR PIV measurements in a metal foam structure. A metal foam sample was scanned using X-ray microtomography and printed with a transparent material using a PolyJet 3D printer. Detailed post-processing methods for the printed model were introduced because conventional methods could not be applied to the small and complex structure. Time-resolved PIV measurements were conducted, and velocity fields were obtained for various regions of interest with inlet laminar flow conditions.

The fluid flow entering the foam configuration was very complicated. The pore network formed a jet; thereafter, the flow was divided and combined. Interflowing jets disturbed shear layers created by other jets. A significant transverse velocity near 0.23 $\langle v\rangle /U_{b}$ was generated, which is in agreement with Onstad et al. [21], who state that significant transverse movement may happen even in the laminar flow inside of metal foams.

The span-wise vorticity evolution in the foam configuration was investigated. The span-wise vorticity inside the metal foam was predicted to increase linearly along the stream-wise direction and show a similar tendency to the integral time/length scale at *z* = 0 D. The span-wise vorticity demonstrated significant oscillation because of complexity of the flow movement inside the foam configuration, and the fluctuation was visualized by the FTLE method. It is believed that this turbulent characteristic was caused by the interaction of jets that have different flow directions inside the metal foam structure. This was expected to enhance the heat and mass transfer inside the metal foam. This study was performed for a Reynolds number and a foam geometry. In addition, channel size was small; therefore, wall effects must be considered. Therefore, there are open doors for future studies on flow features employing different Reynolds numbers and different metal foam arrangements.

## Figures and Tables

**Table 1:** Geometrical characteristics of utilized foam.

Porosity (ε)	$\mathrm{Strut}\;\mathrm{Diameter}\;(d_{s})$	$\mathrm{Pore}\;\mathrm{Diameter}\;(d_{p})$	$\mathrm{Cell}\;\mathrm{Diameter}\;(d_{c})$
0.92	0.8 ± 0.06 mm	3.9 ± 0.33 mm	9.02 ± 1.06 mm

## Data Availability

The data presented in this study are available on request from the corresponding author.
